# Analysis of AlphaMissense data in different protein groups and structural context

**DOI:** 10.1038/s41597-024-03327-8

**Published:** 2024-05-14

**Authors:** Hedvig Tordai, Odalys Torres, Máté Csepi, Rita Padányi, Gergely L. Lukács, Tamás Hegedűs

**Affiliations:** 1 Institute of Biophysics and Radiation Biology, Semmelweis University, Budapest, Hungary; 2 Department of Physiology and Biochemistry, McGill University, Montréal, QC Canada; 3 HUN-REN-SU Biophysical Virology Research Group, Budapest, Hungary

**Keywords:** Computational biology and bioinformatics, Mutation

## Abstract

Single amino acid substitutions can profoundly affect protein folding, dynamics, and function. The ability to discern between benign and pathogenic substitutions is pivotal for therapeutic interventions and research directions. Given the limitations in experimental examination of these variants, AlphaMissense has emerged as a promising predictor of the pathogenicity of missense variants. Since heterogenous performance on different types of proteins can be expected, we assessed the efficacy of AlphaMissense across several protein groups (e.g. soluble, transmembrane, and mitochondrial proteins) and regions (e.g. intramembrane, membrane interacting, and high confidence AlphaFold segments) using ClinVar data for validation. Our comprehensive evaluation showed that AlphaMissense delivers outstanding performance, with MCC scores predominantly between 0.6 and 0.74. We observed low performance on disordered datasets and ClinVar data related to the CFTR ABC protein. However, a superior performance was shown when benchmarked against the high quality CFTR2 database. Our results with CFTR emphasizes AlphaMissense’s potential in pinpointing functional hot spots, with its performance likely surpassing benchmarks calculated from ClinVar and ProteinGym datasets.

## Introduction

In both the medical field and the broader realm of biology, understanding the pathogenicity of mutations holds high significance^1,2^. Pathogenic mutations disrupt the normal function of genes, leading to multiple diseases and medical conditions. From the early onset of genetic disorders in infants to the development of complex diseases in adults, the transformative power of a single nucleotide change can be profound. Discerning between benign and pathogenic mutations can influence diagnostic accuracy, guide therapeutic interventions, and inform prognosis^3^. Therefore, reliable tools and methodologies to predict and understand mutation impact are essential.

Prior to the advent of more advanced genetic analytical tools, several algorithms emerged as standard bearers in predicting the potential impact of mutations, such as PROVEAN, PolyPhen-2, and SIFT. PROVEAN (Protein Variation Effect Analyzer) offers predictions based on the alignment of homologous protein sequences. Meanwhile, PolyPhen-2 (Polymorphism Phenotyping v2) employs a combination of sequence and structural information to classify variants as benign or probably damaging^4^. SIFT (Sorting Intolerant From Tolerant) operates by considering the degree of conservation of amino acid residues in sequence alignments derived from closely related sequences to predict whether an amino acid substitution affects protein function^5^. While these tools have undeniably advanced our understanding of mutation pathogenicity, they also underscore the complexity of the task and highlight the need for continuous refinement in the face of rapidly accumulating genomic data. Newer tools for evaluating the pathogenicity of missense mutations were created. MVP (Missense Variant Pathogenicity prediction) has gained attention for its sophisticated integration of multiple features related to genetic variation^6^. MetaSVM is an ensemble method that merges the outputs of various tools using support vector machines to consolidate pathogenicity prediction^7^. M-CAP (Mendelian Clinically Applicable Pathogenicity) stands out for its high specificity in distinguishing disease-associated variants from neutral ones^8^. VESPA, the Variant Effect Scoring Prediction Algorithm, is based on embeddings of a protein language model, which captures nuanced relationships between amino acid residues, allowing for a more refined and context-aware prediction of variant impacts^9^.

AlphaMissense machine learning, developed recently by DeepMind, can predict the pathogenicity of missense variants and stands at the frontier of missense variant pathogenicity prediction^10^. Importantly, it leverages the structural prediction capabilities of AlphaFold^11^ to analyze these variants. To potentially enhance the precision of missense variant pathogenicity insights, AlphaMissense evolved the field by merging sophisticated machine learning with structural biology. Moreover, AlphaMissense aims to tackle the challenge of interpreting the vast number of missense variants in the human genome, many of which have unclear clinical significance. It holds the promise of revolutionizing the understanding and diagnosis of genetic diseases by classifying missense variants as likely benign or likely pathogenic^10^.

While the conception of AlphaMissense represents a commendable stride, defined by its intricate design and advanced methodologies, there remain gaps in our understanding of its performance on selected groups of proteins or individual proteins. In particular, a pivotal concern arises from the specificities of its missense mutation predictions and the limited accessibility to its dataset. Whereas there are initiatives to make the data accessible through R and Python tools^12–16^, these require a certain level of computational skills, thus significantly restricting the user base. Addressing these voids, we assessed AlphaMissense performance on different datasets using ClinVar data.

## Results

### Performance of AlphaMissense across diverse protein groups in relation to ClinVar data

The performance of AlphaMissense may exhibit variability across different protein types, necessitating careful scrutiny when analyzing target proteins. We evaluated AlphaMissense’s efficiency across a range of protein groups, choosing single nucleotide variants from ClinVar as our benchmark. While ClinVar is a valuable resource, it has its shortcomings. For instance, it may disproportionately represent genes under intensive study while underrepresenting highly pathogenic mutations due to the fact that individuals harboring them might not survive to birth. Additionally, heterozygotes also make it challenging to draw conclusions about the effects of mutations. For our analysis, we juxtaposed all benign and pathogenic missense mutations rated with at least one star in ClinVar against AlphaMissense predictions for proteins in our datasets. Only genes with corresponding ClinVar entries were considered. Subsequently, we derived precision (position predictive value, PPV), recall (true positive rate, TPR), F1 score, aucROC, and Matthew’s Correlation Coefficient (MCC) (Table 1). In general, the calculated statistical measures were high for all the groups studied. Most importantly, MCC exceeded 0.6 for all but two groups, with low values possibly stemming from sparse input data for MemMoRFs and compromised ClinVar data quality, especially for CFTR. We also determined the frequency of likely benign and pathogenic mutations in ClinVar relative to protein length (Table 1).

**Table 1:** AlphaMissense performance on proteins sets, benchmarked with ClinVar.

	n(protein)	n(mutation)	PPV	TPR	F1	aucROC	MCC	f(CV,benign)	f(CV,pathogenic)	f(AM,benign)	f(AM,pathogenic)
ALL	11,486	107,681	0.829	0.776	0.802	0.870	0.697	0.009	0.005	3.425	1.713
MITO	299	2,970	0.804	0.874	0.837	0.888	0.702	0.011	0.010	3.085	2.039
HK	1,011	8,329	0.878	0.801	0.838	0.861	0.714	0.008	0.006	2.899	2.285
SOL	7,938	73,816	0.831	0.746	0.786	0.867	0.686	0.009	0.004	3.484	1.755
IBS	138	55	0.895	0.944	0.919	0.890	0.755	0.340	0.760	1.800	3.880
MemMoRF	35	21	0.462	1.000	0.632	0.846	0.496	0.381	0.619	2.286	2.619
HTP85	1,653	16,015	0.815	0.855	0.834	0.872	0.704	0.009	0.007	3.352	1.762
HTP85 – TM	1,653	1,997	0.895	0.900	0.898	0.872	0.745	0.453	0.683	2.127	3.045
HTP85 – nonTM	1,653	14,018	0.799	0.845	0.822	0.870	0.694	0.623	0.471	3.336	1.881
GPCR	299	1,696	0.802	0.651	0.719	0.871	0.631	0.009	0.003	3.573	1.647
ABC	42	1,557	0.770	0.948	0.850	0.882	0.646	0.009	0.019	3.370	1.787
CFTR	1	207	0.884	1.000	0.939	0.975	0.478	0.005	0.134	3.289	1.776
CFTR (CFTR2)	1	119	0.961	0.961	0.961	0.852	0.725	0.011	0.069	3.289	1.776
lowAF	12	327	0.828	0.358	0.500	0.831	0.481	0.022	0.002	2.466	1.952
lowAF-pLDDT50	12	126	0.828	0.571	0.676	0.819	0.573	0.010	0.003	1.843	2.595
SOL-pLDDT50	7,938	44,045	0.832	0.785	0.808	0.843	0.660	0.007	0.005	2.825	2.333

n(protein): number of proteins with associated ClinVar entries, n(mutations): number of missense mutations from ClinVar SNVss with at least one star, for the given set of proteins, PPV: positive predictive value, TPR: true positive rate, aucROC: Area Under the Receiver Operating Characteristic Curve, MCC: Matthews’s correlation coefficient, f(CV|AM, benign|pathogenic): the number of benign and pathogenic missense mutations from ClinVar (CV) SNV data and from AlphaMissense (AM) predictions was normalized to the number of amino acids (summed length of proteins) for each protein set, MITO: mitochondrial, HK: housekeeping, SOL: soluble, IBS: interfacial binding site, HTP85: Proteins in the Human Transmembrane Proteome with at least a confidence score of 85, TM: transmembrane region only, CFTR2: CFTR2 database entries used for comparison, lowAF: low quality AlphaFold structures, -pLDDT50: without residues with a pLDDT score lower than 50.

Our initial analysis centered on mitochondrial proteins of bacterial origin. Given the unique sequence attributes of these proteins, prediction biases were anticipated. Intriguingly, the pathogenic variation frequency for these proteins was higher than that of the entire human protein ensemble. The important cellular function of these proteins in energy balance might hint their role as housekeeping genes. Drawing from a specific database (https://housekeeping.unicamp.br)^17^, we cross-referenced 1,011 housekeeping genes with 299 mitochondrial genes from our collection and only a modest overlap of 98 genes was observed. The anticipated elevation in pathogenic mutation frequency was evident in the housekeeping gene dataset.

**Fig. 1:**
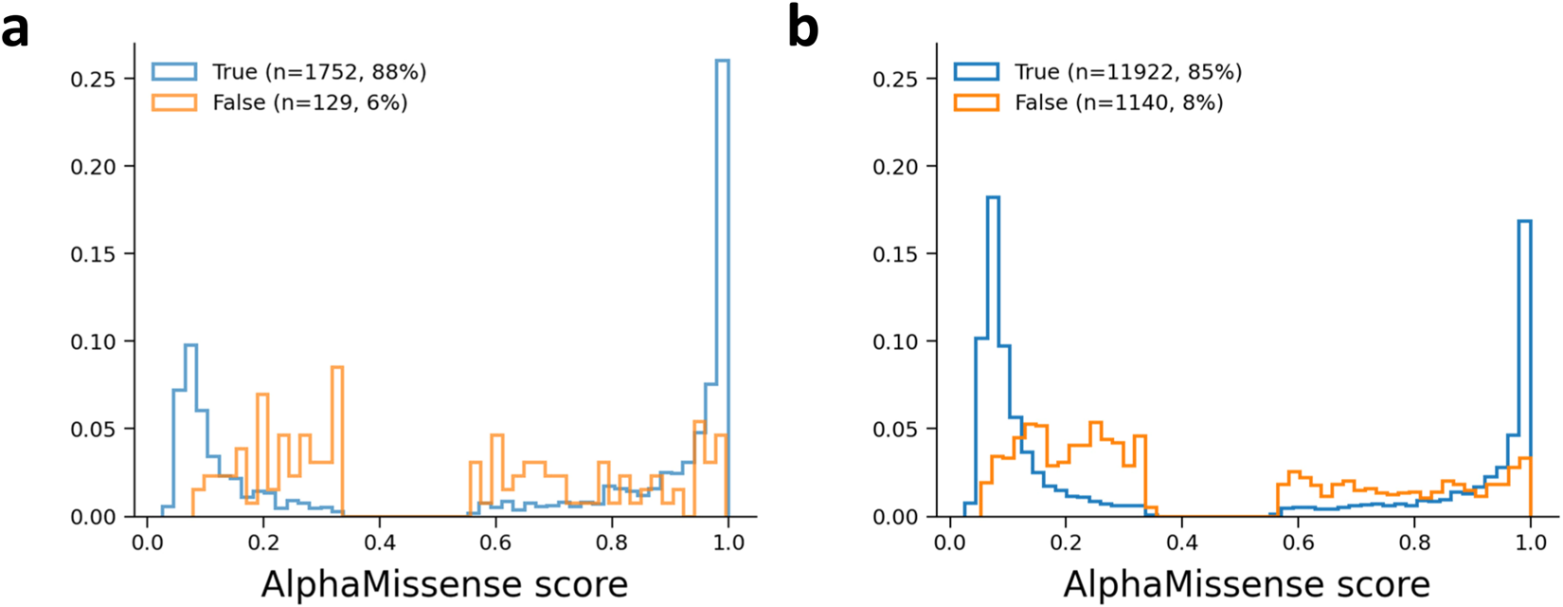
Distribution of AlphaMissense predictions in transmembrane (**a**) and soluble regions (**b**) of TM proteins. Transmembrane and soluble parts were determined for HTP entries with a confidence score higher than 85. Benign and pathogenic AlphaMissense predictions for SNVs present in ClinVar were collected and split into true and false categories for plotting. Ambiguous AlphaMissense predictions (6% and 7% for TM and soluble regions, respectively) were not included.

Mutation frequencies and AlphaMissense efficiency on transmembrane (TM) proteins were also assessed. We segregated residues into TM and non-TM subsets using the Human Transmembrane Proteome database^18^. Counterintuitively, AlphaMissense performed better on TM regions (88% correct and 6% failed predictions versus 85% and 8% for soluble regions, respectively; Table 1 and Fig. 1a,b). This is unexpected, since hydrophobicity reduces sequence variance thus evolutionary insights from sequence alignments. However, the spatial constraints of transmembrane domains lacking intrinsically disordered regions might boost the AlphaFold-based AlphaMissense predictions^19^. Remarkably, pathogenic mutations were more prevalent in TM domains than benign ones (Table 1).

Then we focused on specific membrane protein subsets. While a surge in pathogenic mutations for GPCRs in ClinVar was anticipated, this was not observed. In contrast, ABC proteins manifested elevated pathogenic mutation frequencies in the ClinVar database. Such disparities might be the result of the disease-associated specific protein classes or research biases. Importantly, type and quality of data can profoundly impact these types of analyses. For instance, when juxtaposing AlphaMissense’s predictions against ClinVar data for the CFTR/ABCC7 protein, benign mutations were infrequent, whereas pathogenic mutations predominated. The MCC for CFTR ClinVar/AlphaMissesnse comparison was low (0.478).

Membrane-interacting protein residues were also investigated. One dataset included interfacial binding site (IBS) residues^20^ while the other contained membrane molecular recognition features (MemMoRFs; lipid-interacting disordered regions)^21^. For IBS residues, pathogenic mutations were approximately twice as frequent as benign ones (0.760 vs. 0.340), likely reflecting the functional significance of these residues. Similar trends were evident for the MemMoRF set, although it’s crucial to recognize the limited sample size for this category that might explain the diminished MCC when comparing ClinVar and AlphaMissense outcomes. Moreover, the intrinsic disorder and low sequence conservation of these regions might also influence AlphaMissense’s predictive power on these proteins^10^.

Finally, the potential source of low MCC values were investigated. In the case of CFTR, we tested AlphaMissense predictions against a gold standard CFTR mutation database, CFTR2 (The Clinical and Functional TRanslation of CFTR (CFTR2); available at http://cftr2.org). The CFTR2 database exhibited benign mutation frequencies comparable to other groups but a marked increase in pathogenic mutations. The calculated MCC with this benchmark set was one of the highest (0.725) compared to any of the other protein groups. We assumed that the very low MCC for MemMoRF groups may have caused by the high prevalence of disordered residues in these proteins. Because of the small size of this dataset we tested this possibility on soluble proteins, by excluding those residues from the calculations, which residues exhibit a pLDDT score lower than 50 in AlphaFold structures as a proxy for intrinsically disordered regions^22^. A small increase was observed for PPV, TPR, and F1, but not for rocAUC and MCC values (SOL-pLDDT50 in Table 1) when compared to all soluble proteins. Therefore, we assumed that low results of proteins with MemMoRF may have arisen from the AlphaFold’s capabilities for predicting their structures, since the MemMoRF containing protein set involve several single-pass, bitopic transmembrane proteins. Therefore, we indirectly investigated this possibility, and used a transmembrane protein set with failed AlphaFold predictions^23^, which group of proteins resulted also very low MCC scores (lowAF in Table 1). Interestingly, excluding residues with a pLDDT score lower than 50 (lowAF-pLDDT50 in Table 1) increased the TPR, F1, and MCC scores. The latter score for this set became 0.573.

### Variability in AlphaMissense predictions across different groups of proteins

The observed differences in True Positive Rate (TPR) and F1 scores implied that the distribution of benign and pathogenic mutations is not uniform across protein groups. To gain a deeper insight and understand AlphaMissense’s predictive properties, we investigated the frequency and distribution of its SNV predictions across various protein categories (Table 1). Typically, benign mutations were more frequent, with values hovering between 3 to 3.5, as opposed to pathogenic mutations, which ranged from approx. 1.5 to 2. Given that AlphaMissense predictions cover all possible missense mutations, not biased by human issues, it is reasonable to deduce that only about 30–35% of the possible human missense mutations are pathogenic. A few of our protein sets deviated from this trend. Housekeeping genes displayed slightly lower benign and higher pathogenic mutation frequencies. Both the IBS dataset and the transmembrane regions of transmembrane proteins demonstrated a large reduction in benign and an increase in pathogenic mutation frequencies. This elevated pathogenic frequency in the latter two datasets likely stems from the inclusion of functionally critical sites, which are more susceptible to mutations.

**Fig. 2:**
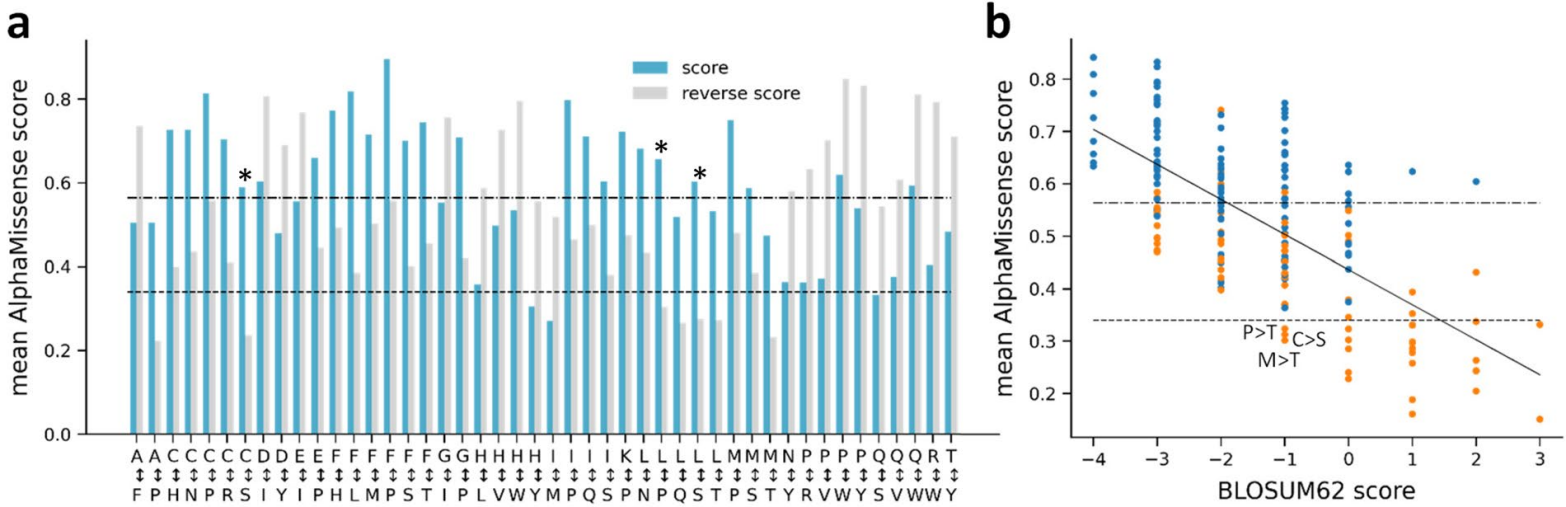
Symmetries of AlphaMissense amino acid substitutions. (**a**) Mean AlphaMissense scores for variations, which display a minimum score difference of 0.2 when compared to the reverse amino acid change. Asterisks mark those changes which get the opposite label (benign/pathogenic) in the case of reverse change. (**b**) Mean AlphaMissence scores for each variation grouped by their BLOSUM62 score. Dashed and dashed dotted lines indicate the cutoffs of the ambiguous AlphaMissense predictions. Solid back line was fitted (r = −0.678, p = 6.39 × 10^−27^). Orange circles: amino acid substitutions possible with single nucleotide change; blue circles: all other substitutions.

We next examined whether the reverse mutations demonstrated similar average AlphaMissense scores. For each variation, we calculated the mean scores and paired them with their reverse counterpart for visualization. We highlighted variation pairs that showed a difference of at least 0.2 in their average scores (Fig. 2a). The pathogenicity labels of three pairs are changed from pathogenic to benign (highlighted by asterisks). The contrasting mean values of the Cys/Ser mutation, categorized as likely-pathogenic, and the Ser/Cys, which is deemed likely-benign, can be rationalized based on amino acid properties and structural implications. Cysteine plays a pivotal structural role, particularly in forming disulfide bridges. In a simplified form, this makes the replacement of Serine with Cysteine more tolerable than the other way around, as Serine cannot replicate Cysteine’s capability in forming disulfide bridges. Accordingly, Cys/Ser pathogenic mutation frequency (0.011) is 5.5 times higher than Ser/Cys pathogenic frequency (0.002) in the ClinVar dataset. The asymmetry of the Leu/Pro replacement can be understood as Pro restricts the available conformational space. The greater disruptiveness of the Leu/Ser replacement compared to Ser/Leu can be attributed to the structural importance of the hydrophobic Leucine, which has a high alpha-helix propensity, in contrast to the hydrophilic Serine that often occurs on protein surfaces^24^.

We also analyzed how the mean scores of all variations correlated with the symmetric BLOSUM62 matrix, a representation derived from amino acid substitution frequencies based on sequence alignments. BLOSUM62 and mean AlphaMissense scores calculated from all possible amino acid substitutions correlated well (correlation coefficient: −0.678, p = 6.39 × 10^−27^, Fig. 2b). Interestingly, numerous average scores for less favorable substitutions fell below the likely-pathogenic threshold set by AlphaMissense. This trend may arise from the higher ratio of variations predicted as likely-benign. Notably, the averages for Cys/Ser, Pro/Thr, and the Met/Thr variations, which have a BLOSUM62 substitution score of −1, lie slightly below 0.34, placing them in the likely-benign category (Fig. 2b).

### Analyzing functional hotspots using AlphaMissense - CFTR as an example

**Fig. 3:**
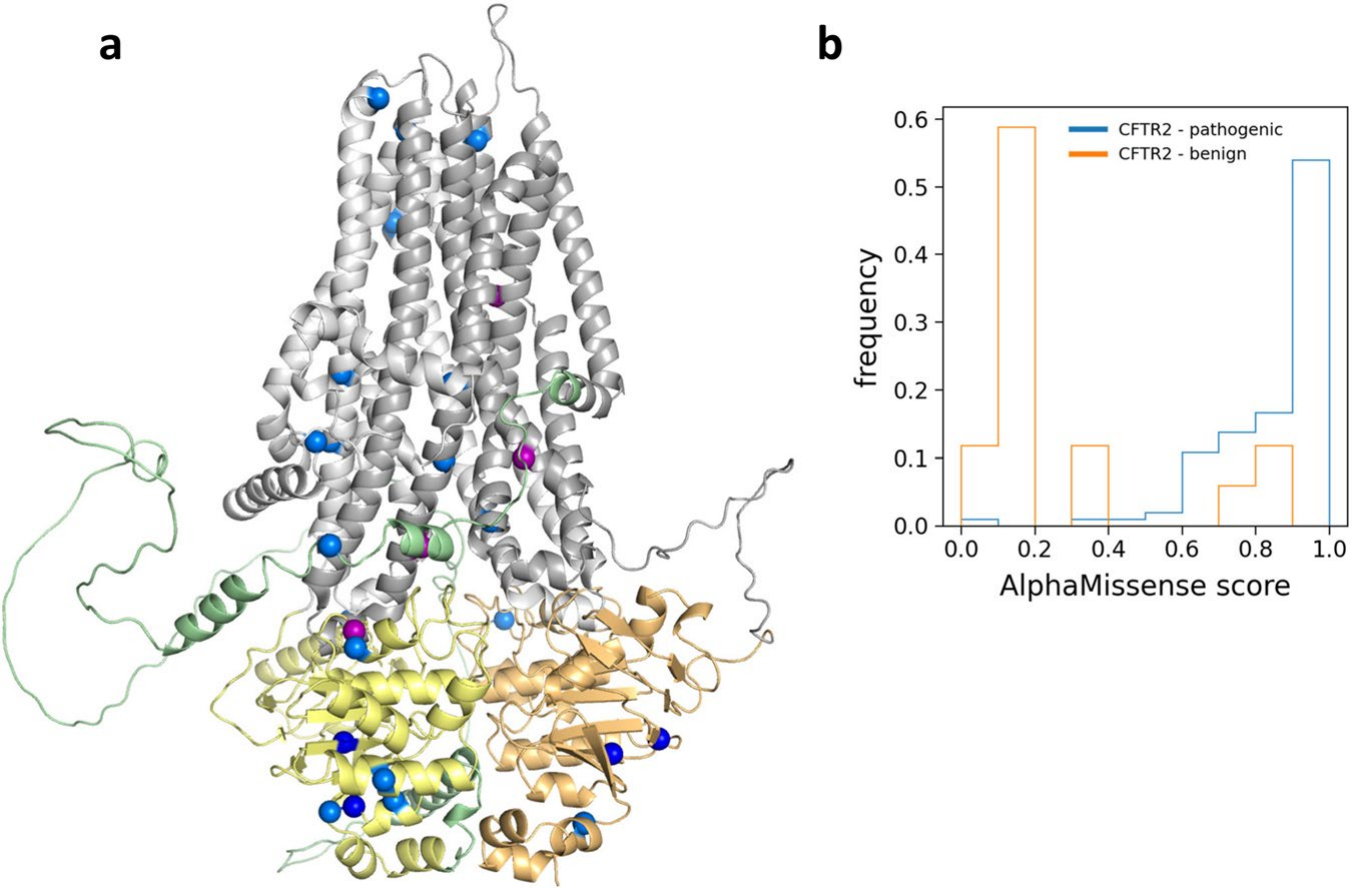
AlphaMissense predictions for CFTR. (**a**) False predictions are shown in the context of the structure (AF-P13569-F1-AM_v4). Light gray: TMD1; yellow: NBD1; gray: TMD2; orange: NBD2; blue spheres: false positive against mutations from ClinVar; dark blue spheres: false positive against mutations from both ClinVar and CFTR2 databases; purple spheres: false negative from CFTR2. (**b**) Histograms of AlphaMissense scores for benign (n = 20) and pathogenic (n = 102) mutations from the CFTR2 database.

We assessed the AlphaMissense predictions for the CFTR protein, which attracted substantial attention within the scientific community, primarily because of its association with cystic fibrosis^25^. For our study, we relied on the CFTR2 database (CFTR2_7April2023.xlsx, https://cftr2.org) to annotate mutations. Impressively, out of the 102 pathogenic and 20 benign mutations listed in the CFTR2 database, AlphaMissense mispredicted only four pathogenic (I601F, A613T, I1234V, and V1240G with scores 0.49, 0.39, 0.08, and 0.5637) and four benign (F508C, L997F, T1053I, and R1162L with scores 0.87, 0.74, 0.35, and 0.89) mutations to the opposite or ambiguous category. Performance metrics for AlphaMissence on CFTR against ClinVar and CFTR2 databases are listed in Table 1 and corresponding false predictions are shown in Fig. 3a utilizing the AlphaFold-predicted structure (AF-P13569-F1-AM_v4)^26^, demonstrating no clusterization of false predictions in specific structural areas, such as interfaces or ATP binding sites. The particular AlphaMissense scores of the 122 values for the CFTR2 mutations are visualized in Fig. 3b.

**Fig. 4:**
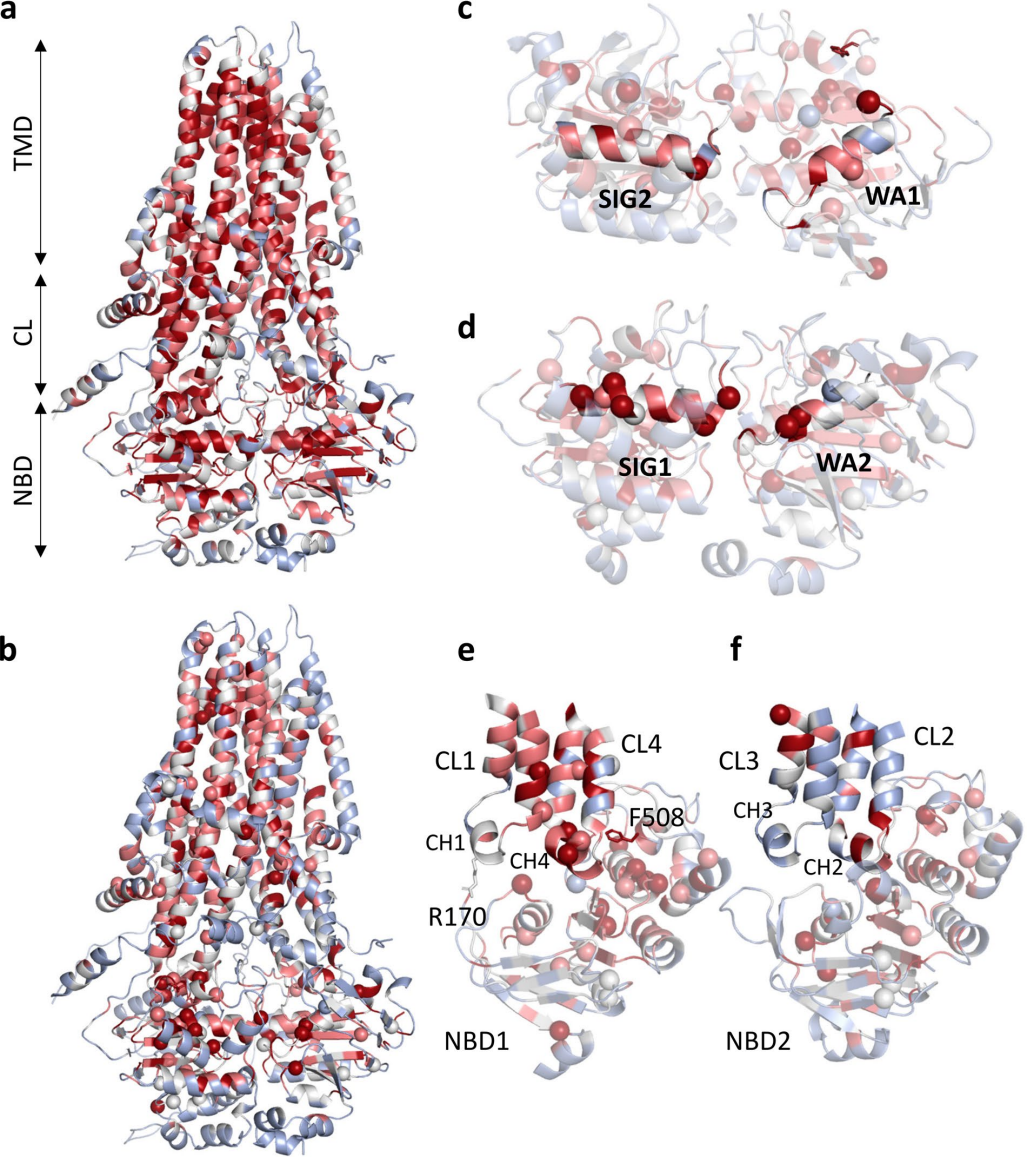
Distribution of AlphaMissense scores for CFTR. (**a**) AlphaFold structure of CFTR (AF-P13569-F1-AM_v4) colored by mean AlphaMissense scores calculated for all possible amino acid changes. (**b**) The same structure was colored by mean AlphaMissense scores calculated for SNVs. Blue: 0–0.340, gray: 0.340–0.564, pink: 0.564–0.780, red: 0.78–1. Spheres represent pathogenic mutations from CFTR2. (**c,d**) The degenerate, non-catalytic ATP-binding Site-1 and catalytic Site-2. Residues 461–472 and 1346–1362 were highlighted for Site-1 structural elements and residues 548–564 and 1247–1258 for Site-2. (**e,f**) Pathogenic mutations and mean scores at the NBD/TMD interfaces. TMD: transmembrane domains, ICL: intracellular loops, NBD: nucleotide binding domains, CL: cytoplasmic loops, sticks: F508 and R170. Coloring scheme of all structures is the same.

For spatial representation of these mutations we used the AlphaFold-predicted CFTR structure colored according the mean AlphaMissense score calculated for SNVs, since multiple nucleotide changes result in more pathogenic amino acid substitutions (Fig. 2b) and mask valuable information (Fig. 4a,b). The ATP binding sites of CFTR, especially, warrant attention. The formation of an ATP binding site is an intricate interplay between one Walker A motif from a Nucleotide Binding Domain (NBD) and a signature motif from the opposite NBD. In comparison to the functional site-2, both the count of CFTR2-sourced mutations and the AlphaMissense scores were observed to be lesser at the site1 (15 versus 3 and 0.584 versus 0.493, respectively; Fig. 4b,c), which site is degenerate, rendering it incapable of ATP hydrolysis^27^. The difference in the mean AlphaMissense scores decreased (0.725 versus 0.675) when calculated not only from possible SNVs but from all amino acid variations. The structural landscape around the F508 residue provides more insight. The CH4 coupling helix, which interacts with the F508 residue, presents a greater number of both predicted and CFTR2-based mutations in comparison to CH2, which is a structural counterpart of CH4 (Fig. 4d,e). No CFTR2 mutations are present in the other coupling helices. CH1, 2, 3, and 4 mean AlphaMissense scores are 0.336, 0.411, 0.136, and 0.648, respectively (0.478, 0.598, 0.237, and 0.773 when calculated from all possible amino acid variations). Interestingly, CH1 was found to be devoid of CFTR2 mutations, but *in vitro* experiments in this region revealed that the R170G mutation, which has a likely-benign AlphaMissense label, impairs the domain-domain assembly and would be pathogenic if harbored by an individual^28^.

The F508 residue is not only an epicenter for deleterious mutations but has also been extensively researched. While CFTR2 lists no additional pathogenic mutations for this residue, a range of experimental works have delved into substituting the Phe with all the other nineteen possible amino acids to discern the impacts on the functional expression of CFTR^29^. All F508 substitution were predicted as likely pathogenic in the AlphaMissense dataset. However, experimental data suggests that apart from the F508C variant the F508V mutation might also be functionally permissive^29^, deviating from AlphaMissense’s likely-pathogenic prediction. Two other variants, labeled as “unknown” or of “varying significance” in the CFTR2 database, show discrepancies between *in vitro* experiments and AlphaMissense predictions. Specifically, the F1052V mutation, predicted by AlphaMissense as likely-pathogenic, demonstrates a functional expression, with 57% mature protein form and 60% functionality relative to the wild type^30^. Conversely, the S912L variant, predicted as benign, appears to be a potential false negative AM prediction. This was based on displayed CF phenotypes in individuals with S912L CFTR^31^ which may be explained by its substantially reduced function, at 16% of the wild type, despite an expression level nearly on par at 92% relative to the wild type^30^. However, earlier research suggests that the S912L variant should be viewed as neutral in isolation, and highlights how complex alleles contribute to the broad phenotypic variability seen in CF^32,33^.

## Discussion

We embarked on an in-depth analysis of AlphaMissense predictions, ranging from broad protein groups down to the individual CFTR protein. Our objective was to gain insights that would aid the interpretation of predictions for specific target proteins, since heterogeneous performance on different protein groups can be expected. For benchmarking purposes, we turned to ClinVar, given its substantial repository of curated and reviewed entries. Remarkably, AlphaMissense exhibited consistent performance across various protein categories, evidenced by an MCC value exceeding 0.6 (Table 1). While these falsified expectations for degraded performance in the case of some protein groups, exceptions arose in scenarios where either the volume of benchmark data was sparse or when the quality of the data was lower. These cases included MemMoRFs and ClinVar’s CFTR data, respectively. Our results indicate AlphaMissense performing well when comparing to the CFTR2 database and suggest that AlphaMissense performance likely performs better than expected based on benchmarks calculated from ClinVar. Our assessment based on CFTR2 is in contrast with the study of McDonald *et al*.^31^, whose differences likely arise from our exclusion of entries with unknown consequences and ambiguous AlphaMissense predictions. The discrepancies observed, like the S912L CFTR mutation^30–33^, between AlphaMissense predictions and studies on CFTR are not unexpected, especially when the mutations in question are part of complex alleles in cystic fibrosis or other diseases. We also emphasize that AlphaFold’s pLDDT scores can provide insights into AlphaMissense performance as the quality of the structures may further indicate the reliability of AlphaMissense predictions (lowAF in Table 1).

Both within ClinVar and the AlphaMissense SNV predictions, benign mutations typically outnumbered their pathogenic counterparts by a factor of approximately two, in several protein groups. Intriguing deviations from this trend were noted in groups such as mitochondrial proteins, housekeeping genes, transmembrane regions of membrane proteins, and IBS residues that pattern aligns with expectations. The IBS dataset, with its notably high pathogenic frequency, exclusively contains functional positions (Table 1). The pathogenicity of CFTR coupling helices were also predicted with remarkable congruency with CFTR2 data (Fig. 4d,e). These observations accentuate the potential of AlphaMissense predictions as a valuable tool for aiding the identification of functionally crucial sites. To facilitate hotspot detection and access to AlphaMissense data, we established a dedicated web resource available at https://alphamissense.hegelab.org, which also provides structure files with mapped AlphaMissense scores for visualization, e.g. in PyMOL with our coloring plugin *coloram.py*, for facilitating local analysis^26^. These enhancements crucially aid in mutational hotspot detection, paving the way for more detailed and user-friendly analyses.

## Methods

### Datasets

The primary AlphaMissence datasets, AlphaMissense_hg38.tsv.gz and AlphaMissense_aa_substitutions.tsv.gz, was sourced from Zenodo (10.5281/zenodo.8208688)^34^. This data contains all predictions with all possible missense variations in the human proteome. For our data analysis, we employed PostgreSQL 12 (https://www.postgresql.org) and Python scripts, which are accessible on Zenodo (10.5281/zenodo.10255502)^26^; refer to README.md and script help options for guidance). We used the load.py script to input data from AlphaMissense_hg38.tsv into the database. To cross-reference ClinVar and UniProt IDs, we executed ‘load_amnames.py’ (using all_acc.pkl file from the clinvar/getids.ipynb IPython notebook and ‘clinvar_result.txt’). Average AlphaMissense scores for each residue were calculated and saved in the database via the load_amspots.py script.

Missense data was retrieved from ClinVar^35^ as of 26^th^ September 2023 and made available at Zenodo (clinvar_result.txt)^26^. The dataset representing the human proteome was obtained from UniProt Release 2023_04, specifically from the file UP000005640_9606.dat (reference proteomes from https://www.uniprot.org/help/downloads)^36^. This dataset proved instrumental in mapping Ensemble IDs from ClinVar to UniProt accession numbers since the inherent online ID mapping tool at UniProt matched only a very low number of entries.

Human protein structures were downloaded from AlphaFoldDB (version 4; https://alphafold.ebi.ac.uk/download#proteomes-section)^37^. The gen_pdb_occupancy.py script was used to insert the mean AlphaMissense score for each residue into the occupancy and B factor columns of structure files. All of these structures are available at Zenodo as a zip file for bulk download. Individual structure files can be accessed manually or programmatically as https://alphamissense.hegelab.org/pdb/AF-{UNIPROT_ACC}-F1-AM_v4.pdb.

Data for comparing AlphaMissense performance on different groups of proteins presented in Table 1 were collected as follows. Mitochondrial (MITO) Protein Data was procured from MitoCharta (https://www.broadinstitute.org/mitocarta/mitocarta30-inventory-mammalian-mitochondrial-proteins-and-pathways)^38^. The downloaded Human.MitoCarta3.0.xls file was processed with mito/get_accs.ipynb resulting in the list of UniProt ACCs (mito/mito-accs.pkl). Housekeeping (HK) genes were collected from Housekeeping_GenesHuman (https://housekeeping.unicamp.br^17^) using hk/get_accs.ipynb, resulting in hk-accs.pkl. Proteins were considered soluble (SOL; htp/sol-accs.pkl generated by htp/get_accs_sol.py) if they were not listed in the Human Transmembrane Proteome (HTP; https://htp.unitmp.org; htp_all.xml, version d.2.0). The boundaries of membrane regions in transmembrane proteins were sourced from htp_all.xml and filtered to include only entries boasting a quality score greater than 85 to maintain the integrity and accuracy of our analyses^18^ (get_accs_htp85.ipynb produced htp85-accs.pkl). TM (HTP85-TM) and non-TM (HTP85-nonTM) regions were handled by our htp/htp.py library. HTP entries omitted from the TM analysis were not incorporated into the dataset encompassing soluble proteins. Since this criterion resulted in a sparse representation of high-quality predictions for ABC proteins, we supplemented the data with TM boundaries from our ABCM2 database (http://abcm2.hegelab.org; abc-accs.pkl and abcm-tm-boundaries.pkl)^39,40^. GPCR data were downloaded from https://gpcrdb.org/services/receptorlist/ (get_receptorlist.py produced gpcr-accs.pkl output). The CF2 database were accessed for high quality data on CFTR mutations. CFTR2_7April2023.xlsx file was processed with process_cf2.py to generate cf-muts.pkl. IBS data was downloaded as https://github.com/reuter-group/pepr2ds/blob/main/Ressources/datasets/PePr2DS.csv.zip and processed in the select_ibs_residues.ipynb notebook. MemMoRF data was extracted from our database (https://memmorf.hegelab.org, memmorf_extract_20220725.tsv, mmorf-residues.pkl). UniProt identifieres of failed AlphaFold TM protein predictions were manually extracted from Jambrich *et al*.^23^ and stored in a Python list (lowaf-accs.pkl). Datasets with excluded residues with pLDDT lower than 50 (lowAF-pLDDT50 and SOL-pLDDT50) were not generated explicitly. These residues were filtered within the analysis script (see below).

### Analysis

All data analyses were carried out using Python-based tools to ensure flexibility and scalability. To facilitate a lightweight and seamless interaction with the data stored in PostgreSQL 12, we employed the SQLalchemy 2.0.21 library^41^ renowned for its capability to provide a high-level, Pythonic interface to relational databases. Matplotlib 3.7.0 was used for generating plots that delineate various aspects of the data^42^. Structural visualization of proteins was done using PyMOL (version 2.4, Schrödinger, LLC.), a molecular graphics system with an embedded Python interpreter. To bridge the predictions of AlphaMissense with these structures, MDAnalysis 2.4.2 was employed^43^. This Python toolkit allowed us to incorporate the AlphaMissense scores directly into the PDB files, specifically inserting them into both the occupancy and B-factor columns.

The ClinVar entries and AlphaMissense predictions of the above protein groups were compared using ana_clinvar_set.py, ana_clinvar_resi.py, and ana_clinvar_set_plddt.py when full protein sequences, specific residues (e.g. IBS, MemMoRF, and TM residues), and residues with high pLDDT scores were analyzed, respectively. Since aucROC calculation requires not only a contingency table but all the true labels and predictions, aucROC was calculated with separate scripts named calc_*_aucroc.py. The outputs were collected in an Excel table (table1.xlsx).

The AlphaMissense scores were averaged for all possible amino acid changes for each residue in the full dataset using the calc_revfreq.py script (output is stored in aaaa_revfreq.pkl). The AlphaMissense scores were also averaged for pairwise amino acid changes (aaa_freq.pkl) to compare them with the BLOSUM62 substitution matrix. The substitution matrix was taken from the BioPython 1.81 package (https://biopython.org/docs/latest/api/Bio.Align.substitution_matrices.html). The aa_substitutions.ipynb notebook contains the code for analysis including linear regression and plotting the panels of Fig. 1.

Distribution of AlphaMissense scores for CFTR benign and pathogenic variations listed in the CFTR2 database were calculated and plotted (Fig. 2a) with the ana_mutspreds.ipynb notebook. The CFTR structure AF-P13569-F1-AM_v4.pdb was visualized in PyMOL and colored using coloram.py script (Fig. 2b). Residues indicated pathogenic in the ClinVar database are displayed with spheres using the show_clinvar_patho.py script. AlphaMissense mean values referenced in the main text for ATP binding sites (Fig. 2c) and for NBD/TMD interfaces (Fig. 2c).

were calculated using the atpbsites-mean.py and interfaces-mean.py scripts, respectively. ATP binding sites and coupling helices in these panels were highlighted by setting the cartoon_transparency to 0.5 for all other parts of the structure.

## Data Availability

Both input and output data are available at Zenodo (10.5281/zenodo.10023059)^26^. The files are organized into specific directories (e.g. according to datasets) and named in the Methods section and in the README.md file. AFwAM-pdb.tar contains compressed AlphaFold structures with AlphaMissense scores from SNVs in their occupancy and B factor columns. AFwAM-pdb-qb.tar includes structures with scores from SNVs and scores from all possible amino acid replacements in the B factor and occupancy columns, respectively.

## Code availability

Python scripts and IPython notebooks can be accessed at Zenodo (10.5281/zenodo.10023059)^26^. These files also include scripts, which were used to generate the data in Table 1 and to create Figs. 1, 2. They are stored along with data files in the pub.zip archive and listed in detail in the Methods section and the README.md file. The coloram.py script is a PyMOL plugin for coloring structures in PyMOL.
